# Functional basis of ecological divergence in sympatric stickleback

**DOI:** 10.1186/1471-2148-13-277

**Published:** 2013-12-31

**Authors:** Matthew D McGee, Dolph Schluter, Peter C Wainwright

**Affiliations:** 1 Department of Evolution and Ecology, University of California Davis, 1 Shields Avenue, Davis, CA 95616, USA; 2 Biodiversity Research Centre and Zoology Department, University of British Columbia, 2329 W MallVancouver BC V6T 1Z4, Canada

**Keywords:** *Gasterosteus aculeatus*, Functional morphology, Suction feeding, Postglacial fishes, Ecological speciation

## Abstract

**Background:**

The evolution of ecological divergence in closely related species is a key component of adaptive radiation. However, in most examples of adaptive radiation the mechanistic basis of ecological divergence remains unclear. A classic example is seen in the young benthic and limnetic stickleback species pairs of British Columbia. In each pair the benthic species feeds on littoral macroinvertebrates whereas the limnetic feeds on pelagic zooplankton. Previous studies indicate that in both short-term feeding trials and long-term enclosure studies, benthics and limnetics exhibit enhanced performance on their own resource but fare more poorly on the other species’ resource. We examined the functional basis of ecological divergence in the stickleback species pair from Paxton Lake, BC, using biomechanical models of fish feeding applied to morphological traits. We examined the consequences of morphological differences using high speed video of feeding fish.

**Results:**

Benthic stickleback possess morphological traits that predict high suction generation capacity, including greatly hypertrophied epaxial musculature. In contrast, limnetic stickleback possess traits thought to enhance capture of evasive planktonic prey, including greater jaw protrusion than benthics and greater displacement advantage in both the lower jaw-opening lever system and the opercular four-bar linkage. Kinematic data support the expectations from the morphological analysis that limnetic stickleback exhibit faster strikes and greater jaw protrusion than benthic fish, whereas benthics exert greater suction force on attached prey.

**Conclusions:**

We reveal a previously unknown suite of complex morphological traits that affect rapid ecological divergence in sympatric stickleback. These results indicate that postglacial divergence in stickleback involves many functional systems and shows the value of investigating the functional consequences of phenotypic divergence in adaptive radiation.

## Background

Improving our understanding of the process of adaptive radiation requires a more complete understanding of the origin and maintenance of ecological divergence between closely related species [1-4]. The four key properties of adaptive radiation are common ancestry, rapid speciation, phenotype-environment correlations, and trait utility. Shared ancestry is the most commonly tested criterion, typically using a phylogeny with sampling both within the radiation and in its close relatives [5]. Testing for elevated rates of speciation requires temporal information, typically age estimates for newly invaded regions and estimates of divergence times in the phylogeny [6,7]. It is also important to establish the existence of a correlation between the phenotypic traits of species within the radiation and the environments they are found in [8-10]. However, phenotype-environment correlations in the absence of performance data do not necessarily indicate that trait differences play an important ecological role, as measured trait differences may result from correlations with other traits under selection or as a consequence of developmental constraints unrelated to ecology [11-13].

Here we address the final and crucial criterion for adaptive radiation, trait utility, “evidence that traits are useful where they are employed” [4]. Trait utility provides the critical link between phenotype and performance and is required to strengthen inferences of the role of natural selection in producing the radiation. One way to assess trait utility is to carry out manipulative experiments on traits to linking a feature of the phenotype directly to a relevant performance character [14]. Another way, which we adopt here, involves the use of functional models of feeding performance developed in other phylogenetically and morphologically similar species, which allow the calculation of performance from phenotypic data [13,15].

The stickleback species pairs offer an excellent system to test the importance of trait utility in adaptive radiation. In a series of British Columbia lakes created within the last 10,000 years by retreating glaciers, threespine stickleback, *Gasterosteus aculeatus*, have repeatedly diverged into a planktivorous (hereafter “limnetic”) species and a benthic-feeding (hereafter “benthic”) species [16-19]. In most species-pair lakes, benthic and limnetic stickleback are more closely related to each other than they are to ecologically similar forms in nearby lakes [20,21]. Limnetics feed mostly on evasive pelagic calanoid copepods with long strain-sensitive antennae capable of detecting incoming predator attacks, giving the copepod time to escape [22,23]. Benthics feed mostly on non-evasive buried and attached littoral macro-invertebrates that must be detected, then forcibly extracted from their hiding places. In short-term feeding trials, individuals of each species experienced higher prey capture success feeding on their preferred prey than when feeding on the other species’ prey [24,25], suggesting that the trophic apparatus plays a direct role in dietary divergence. In enclosure experiments in native lakes, individuals of each species raised in the appropriate habitat grew faster than when raised in the other species’ habitat [26], and limnetic-benthic hybrids exhibit signs of lower fitness than either parental form in nature [27].

**Figure 1:**
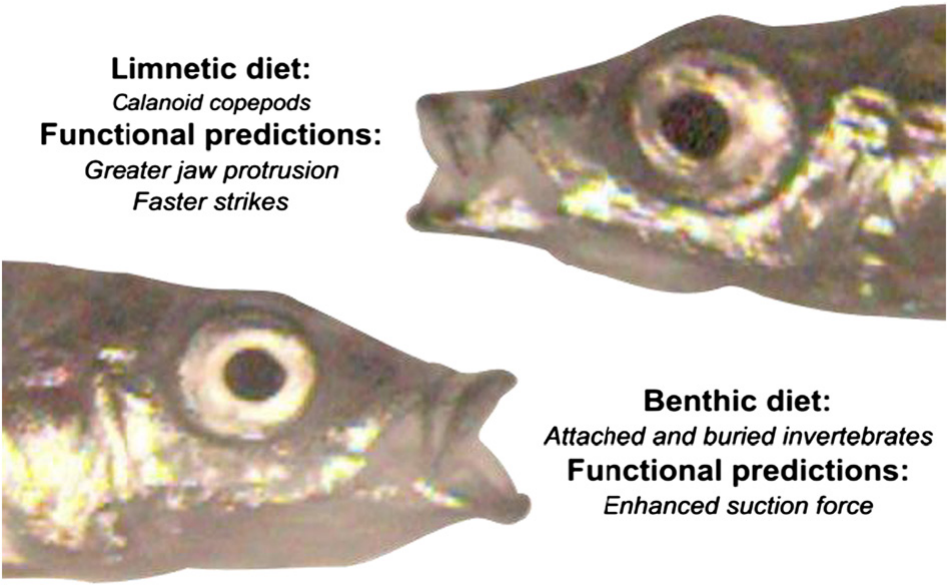
**Limnetic/benthic stickleback diets and associated functional predictions.** Photographs are stills from high-speed feeding kinematics.

Structure and performance associated with the prey capture mechanism may help clarify the functional basis of ecological divergence in the species pairs (Figure 1). Like many teleosts, stickleback are suction-feeding predators that capture prey by expanding the buccal cavity to draw prey items into the mouth. Limnetic stickleback feed on evasive strain-sensitive copepods, so we might reasonably expect limnetics to possess morphology associated with rapid prey capture kinematics [23]. Suction is required to dislodge the buried and attached invertebrates that make up the bulk of benthic diets, suggesting that benthics may possess functional systems adapted to exert higher force on attached prey items [28].

The biomechanics of suction feeding can be quantified using a series of functional models that treat craniofacial bones and muscles as sets of complex levers and linkages. The suction index model predicts the relative morphology potential to produce suction pressure in fish species that use cranial rotation to expand the buccal cavity [15,29]. The opercular four-bar linkage predicts the magnitude of rotation in the articular, the output link, for a set amount of rotation by the interopercule, the input link [30,31]. The jaw lever system predicts the amount of rotation in the fish’s jaw for a given amount of input rotation in the articular [32]. Jaw protrusion refers to the anterior excursion of the ascending process of the premaxilla during mouth opening [33]. In other fishes, these models have accurately predicted patterns of prey use as well as prey capture kinematics *in vivo*. [14,15,30,32].

In this study, we evaluate trait utility by using morphological data and functional models of fish feeding to predict kinematic patterns, then test these predictions by analyzing high-speed films of feeding behavior in limnetic and benthic fish to generate both kinematic and simulated performance data. We then discuss how component trait divergence in the four functional systems affects ecological divergence in the species pair. Our approach deepens our understanding of the mechanisms of adaptive divergence.

## Results

**Figure 2:**
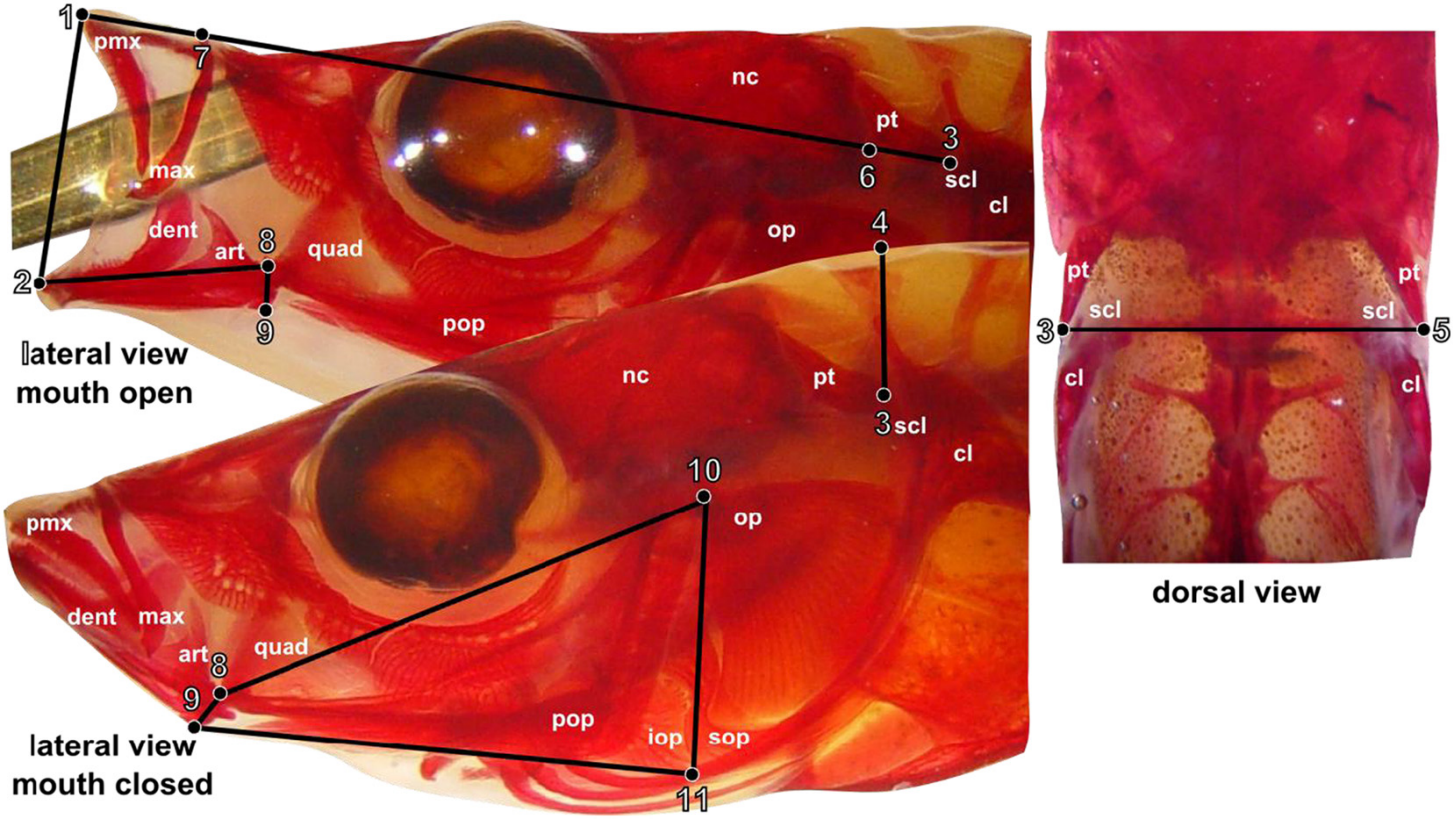
**Morphological components of four functional systems associated with prey capture in percomorph fishes.** Landmarks: (1) anteriormost extent of premaxilla; (2) anteriormost extent of dentary; (3) point of articulation between the supracleithrum and post-temporal; (4) dorsalmost extent of epaxial, measured dorsal to landmark 3; (5) point of articulation between supracleithrum and post-temporal on opposite side of fish, measured in the frontal plane; (6) posteriormost extent of buccal cavity, measured between landmarks 1 and 3; (7) anteriodorsal extent of maxilla; (8) quadrate-articular jaw joint; (9) insertion of the interopercular-articular ligament; (10) opercular joint; (11) posterioventral extent of interopercule. Bone names: pmx = premaxilla, max = maxilla, art = articular, quad = quadrate, pop = preopercule, iop = interopercule, sop = subopercule, op = opercule, pt = post-temporal, scl = supracleithrum, cl = cleithrum, nc = neurocranium.

We uncovered substantial functional and kinematic differences between the two stickleback species. Paxton Lake benthic and limnetic stickleback differ in all four of the functional systems examined in this study: suction index, transmission coefficient of the opercular four-bar, lower jaw opening displacement advantage, and jaw protrusion (Figure 2, Table 1). Jaw protrusion is higher in limnetics, as are opercular four-bar transmission coefficent and lower jaw opening displacement advantage (Table 1). Suction index is higher in benthics (0.017 vs 0.010 in 50 mm fish, p < 0.001). These differences imply that benthics have the capacity to generate higher suction pressure than limnetics, whereas limnetics will have faster jaw movements and greater jaw protrusion during the strike.

**Table 1:** Functional feeding systems and component traits of limnetic and benthic stickleback

***Functional system***	***Landmarks (Figure 2)***	***W (rank-sum test)***	***Mean ± SE, Limnetic***^***1***^	***Mean ± SE, Benthic***^***1***^
*Suction Index*	*1,2,3,4,5,6*	523***	*0.010 ± 0.001*	*0.017 ± 0.001*
*Disp. adv., jaw opening*	*2,8,9*	13***	*5.96 ± 0.01*	*4.82 ± 0.01*
*Opercular four-bar KT*	*8,9,10,11*	98***	*5.75 ± 0.13*	*5.15 ± 0.13*
*Jaw protrusion*	*1,7*	99***	*1.76 ± 0.07 mm*	*1.48 ± 0.07 mm*
*Morphological component traits*	*Landmarks (Figure*2*)*	*W (rank-sum test)*	*Mean ± SE, Limnetic*^*1*^	*Mean ± SE, Benthic*^*1*^
Suction Index:				
*Gape*	*1,2*	*343*	*5.15 ± 0.13 mm*	*5.33 ± 0.13 mm*
*Buccal length*	*1,6*	*204*	*15.05 ± 0.21 mm*	*14.66 ± 0.21 mm*
*Neurocranium outlever*	*1,3*	*292*	*16.55 ± 0.20 mm*	*16.47 ± 0.20 mm*
*Epaxial height*	*3,4*	493***	*1.95 ± 0.08 mm*	*2.35 ± 0.08 mm*
*Epaxial width*	*3, 5*	572***	*5.46 ± 0.09 mm*	*6.38 ± 0.09 mm*
*Disp. adv., jaw opening:*					
*Jaw opening outlever*	*2,8*	*248*	*5.60 ± 0.11 mm*	*5.54 ± 0.11 mm*	
*Jaw opening inlever*^*2*^	*8,9*	568***	*0.94 ± 0.02 mm*	*1.15 ± 0.02 mm*	
Opercular four-bar:					
*Coupler link*	*9,11*	*262*	*6.83 ± 0.10 mm*	*6.86 ± 0.10 mm*	
*Fixed link*	*8,10*	*231*	*9.56 ± 0.12 mm*	*9.44 ± 0.12 mm*	
*Input link*	*10,11*	512 ***	*5.27 ± 0.08 mm*	*5.71 ± 0.08 mm*	
*Output link*^*2*^	*8,9*	568 ***	*0.94 ± 0.02 mm*	*1.15 ± 0.02 mm*	

^1^Trait values calculated using linear regression of SL (set to 50 mm) and ecomorph.

^2^Jaw opening inlever = output link of the opercular four-bar.

***p < 0.001.

Five of the 11 morphological variables were significantly different between species: epaxial height, epaxial width, output link, input link, and opening jaw inlever (Table 1). The greater epaxial width and epaxial height of benthics and the smaller opening jaw inlever of limnetics are consistent with observed functional divergence, because epaxial cross-sectional area increases Suction Index and a smaller opening jaw inlever increases displacement advantage of jaw opening. Also, the input and output link of the opercular four-bar differ between limnetics and benthics (Table 1), in a way that improves force transmission in benthics and velocity transmission in limnetics. Slopes of the relationships between the opercular four-bar fixed link (p < 0.05) and opercular four-bar coupler link (p < 0.05) differed significantly.

As expected from the morphological measurements, our linear mixed model analysis of kinematic data revealed that limnetics exhibit greater jaw protrusion than benthics, and they have shorter times to peak gape, peak lower jaw rotation, and prey capture (Table 2). SL had a significant effect on some of the kinematic variables, including maximum gape, time to peak gape, time to peak cranial rotation, and time to prey capture. By including it as a covariate in our model, the kinematic differences recorded are corrected for size effects. Our mixed-model analysis using Suction Induced Force Field (SIFF) data also indicated that benthics would exert higher maximum force on a simulated attached prey than limnetics (Table 2).

**Table 2:** Kinematic divergence in a stickleback species pair

**Trait**	**pMCMC (SL)**	**pMCMC (ecomorph)**	**Limnetic value†**	**Benthic value†**
**Excursions:**				
Gape	0.0004***	0.53	2.91 mm	2.81 mm
Jaw protrusion	0.87	0.008**	1.35 mm	1.01 mm
Cranial rotation	0.12	0.21	8.72 deg	7.12 deg
Lower jaw rotation	0.18	0.22	25.06 deg	23.31 deg
Strike distance	0.11	0.88	2.87 mm	2.92 mm
**Timings:**				
Gape	0.0034**	0.01*	4.6 ms	8 ms
Jaw protrusion	0.10	0.13	7.8 ms	11.9 ms
Cranial rotation	0.06	0.31	7.3 ms	9.6 ms
Lower jaw rotation	0.04*	0.009**	5.3 ms	9.8 ms
Prey capture	0.04*	0.049*	6.3 ms	9.9 ms
**Forces:**				
Attached prey	0.63	0.045*	2.3 × 10^-4^ N	3.1 × 10^-4^ N

†The ecomorph values were calculated using the fixed effect of SL (set for a 40 mm fish) and the fixed effect of ecomorph from a mixed-effect model for each kinematic trait.

*p < 0.05, **p < 0.01, ***p < 0.001.

## Discussion

Our results reveal a previously unknown suite of complex morphological traits involved in rapid sympatric ecological divergence in a species pair of postglacial fish. Kinematic predictions derived from functional analyses of these morphological traits match observations of high speed prey capture attempts in the plankton-feeding limnetic and the littoral macroinvertebrate-feeding benthic. These results show the value of investigating trait utility for understanding the performance consequences of phenotypic divergence in adaptive radiation.

### Predicted functional differences

Based on our morphological analysis, we predicted large differences between the ecologically differentiated forms in their functional performance when feeding. Few of these differences had been anticipated in previous work on the ecology and morphology of this system. These differences likely contribute to divergent feeding success and growth rate in transplant experiments in the native lakes [26,27,34], and show the value of a functional analysis of morphological differences between species.

Benthics have the potential to generate greater suction pressure and therefore generate greater suction flow speed. Higher suction index values lead to increased suction flow speeds and have been shown to improve performance in computational models of suction feeding on buried and attached prey items [28]. Similar higher suction index values are also observed in benthic stickleback populations in other, independently derived species pairs [35]. The increased suction index values of Paxton benthics are driven mainly by two epaxial traits that differ between limnetics and benthics (Table 1). These hypertrophied epaxial muscles give benthics their distinctive “humped” phenotype [16] and contribute to increased body depth. We suggest that the body depth variation commonly observed between lake-stream stickleback and in recently deglaciated areas is likely connected to variation in the size of the epaxial muscles [36-38].

Oral jaw traits also show a strong pattern of divergence between benthics and limnetics. When the neurocranium is elevated in preserved fish, limnetics exhibit more jaw protrusion than benthics. Zooplanktivorous teleosts often possess high jaw protrusion, which is thought to aid in the capture of strain-sensitive planktonic prey, particularly calanoid copepods [22,23]. The increased morphological jaw protrusion of limnetic fish sets up clear kinematic predictions: limnetics should be able to project their oral jaws farther than benthics during the strike. The opening jaw lever system indicates that limnetics possess more displacement advantage when opening the lower jaw. Assuming equal input velocity, output velocity will be proportional to displacement advantage, implying that limnetics should rotate the lower jaw and open the mouth more rapidly than benthics.

Divergence in the transmission coefficient of the opercular four-bar mirrors divergence in the opening jaw lever system, with limnetics exhibiting a higher transmission coefficient than benthics. This similarity between the opercular four-bar and opening jaw lever likely occurs because both systems share a component trait, the output link/opening jaw inlever (Figure 2, Table 1). In limnetics, an increase in this component trait increases velocity transmission of the opercular four-bar while simultaneously increasing displacement advantage of jaw opening. Divergence in the opercular four-bar transmission coefficient is also driven by an increase in the input link due to dorsoventral expansion of the opercular series in benthic stickleback. Recent work on stickleback opercle shape suggests that dorsoventral variation in the Paxton species pair and across populations is connected to a developmental module that is likely under selection [39,40]. It is likely that recent stickleback opercle shape evolution is a consequence of selection on the opercular four-bar transmission coefficient.

In other teleosts, the opercular four-bar has been less predictive of kinematics than the anterior jaw linkage [30,32], though it clearly is involved in jaw depression since fish with a severed opercular four-bar linkage exhibit disrupted feeding kinematics [41]. Kinematic implications of the differences in four-bar mechanics suggest a similar pattern as the opening jaw lever. The higher transmission coefficient of limnetics predicts that more output rotation is produced for a given input rotation, which should allow limnetic stickleback to open their jaws more rapidly than benthics during a strike.

Complex functional systems, including often diverge in their component traits while converging in their functional outputs, a phenomenon called many-to-one mapping [42]. For example, benthic stickleback from Alaska and British Columbia have independently evolved an increased suction index by modifying different components of the system in each population, resulting in a nearly threefold increase in morphological diversity relative to their anadromous common ancestor [35]. We suggest that future studies of morphological evolution in postglacial fishes are likely to reveal functional solutions similar to those seen in Paxton Lake, even if the individual traits comprising these solutions vary.

### Kinematics

Kinematic data support many of the predictions derived from functional morphology, implying a strong relationship between form and function in this young radiation.

Limnetics have higher maximum jaw protrusion, shorter time to peak gape, shorter time to peak lower jaw opening, and shorter time to prey capture than benthics. All of these traits are expected to improve performance on strain-sensitive prey like calanoid copepods and other crustacean zooplankton, according to simulation studies and live trials with suction-feeding fish species [23,28]. In limnetics, higher speeds of jaw opening and rapid projection of the flow field towards the prey via jaw protrusion both minimize the window of time in which attacked copepods can sense the incoming flow field while simultaneously exposing the prey to a more rapid increase in suction flow speed.

Phenotypic plasticity is thought to play a major role in evolution, and adaptive plasticity has been documented in stickleback and other postglacial fishes [43,44]. Our morphological dataset used wild-caught benthic and limnetic fish, while our kinematic dataset used F1 benthics and limnetics raised in outdoor experimental ponds designed to mimic the natural habitat of Paxton Lake. Phenotypic variation in our morphological dataset is affected by both genetics and environment, whereas fish from the kinematic dataset would have been less influenced by environment. However, despite a potential reduction in environmental influences on phenotype in our kinematic dataset, we see clear differences between benthics and limnetics.

Our simulations suggest that benthics exert higher forces on attached prey items than limnetics do. Many common benthic prey items, such as chironomid larvae, burrow in the substrate or within aquatic plants and must be forcibly extracted once located [45]. Other benthic prey items, like amphipods, can grip or cling to objects in the littoral zone, requiring the predator to dislodge them [46]. Enhanced force generation via increased suction pressure is thus likely to increase the ability of benthic stickleback to capture littoral macroinvertebrates by increasing the proportion of successful strikes produced by the fish.

Understanding the functional consequences of phenotypic divergence is centrally important to studies of adaptation [47]. A careful examination of trait utility can help to separate functionally relevant traits from less relevant shape differences. For example, the distinctive “hump-backed” phenotype of benthic stickleback represents one of the largest shape differences between species [16,17], but the functional consequences have not previously been understood. This phenotype is caused by hypertrophied epaxial muscle posterior to the neurocranium. These enlarged muscles increase the physiological cross-sectional area and therefore force generation of the muscles elevating the neurocranium during a prey capture attempt [15,29]. All else being equal, more forceful epaxial input will result in stronger suction pressure. Enhanced suction pressure is strongly connected with increased performance on attached prey, suggesting that these enlarged muscles may help benthics forage on littoral macro-invertebrates [28]. These results mesh well with previous morphometric work indicating that fish from lakes with a high littoral area and therefore more benthic prey tend to have shapes consistent with hypertrophied epaxial musculature [48-55]. Interestingly, a similar pattern exists in Darwin’s finches, with muscle traits strongly contributing to divergent bite forces between closely-related species [56]. In both finches and stickleback, variation associated with the cross-sectional area of cranial muscles plays a pivotal role, suggesting that variation in the sizes and shapes of muscles can be as important as changes in the structure of hard bony elements [57-60].

Morphological and kinematic gape data indicate that, contrary to previous studies, size of the open mouth differs little between benthics and limnetics after body size correction, particularly when compared to changes observed in the epaxial muscles. Benthic and limnetic stickleback were previously thought to differ in mouth size, with benthics possessing a larger closed-mouth gape width [17]. Paxton benthics and limnetics do differ in the width of the closed mouth, but teleost mouths are highly kinetic and change shape over the course of a prey capture attempt [29,61]. Our results indicating a lack of divergence in mouth size make sense in the context of benthic suction feeding, as a larger mouth would increase the area of the fish’s buccal cavity, reducing the suction pressure it could exert on attached prey.

Studies of trophic morphology in postglacial radiations have mostly focused on the gill rakers, which are thought to enable zooplanktivorous limnetic ecomorphs to retain small prey items obtained through suction feeding [62,63]. Our kinematic and morphological results are consistent with divergence in gill raker morphology, with limnetic fish using rapid strikes to ingest small zooplankton, then using the rakers to prevent escape from the fish’s buccal cavity. The function of large raker spacing in benthic stickleback has yet to be established, and could be associated with the need to sort food items from benthic debris after a strike, or it could simply be a function of the larger prey sizes consumed by benthics [16,64]. Though gill rakers are certainly a functionally important trait [65,66], experimental studies of gill raker function (eg. surgical removal of the rakers) have focused on specialized phytoplanktivorous oreochromine cichlids, rather than species with zooplanktivorous diets similar to postglacial limnetic fishes [67,68].

Postglacial radiations also differ in many ecologically important traits aside from trophic morphology [69,70]. Limnetic stickleback are often more exposed to predation than benthics, favoring divergence in cryptic coloration and defensive weaponry [71-73]. Differences in structural complexity of the habitat can lead to divergence in maneuverability, sustained swimming, and spatial processing [74-78]. Traits related to searching for prey can also differ, including vision and neuromast patterning [79,80]. The large number of potential phenotypic differences emerging between young stickleback species pairs suggests that further study of integration [81] in the genetic and phenotypic architecture of postglacial radiation is likely to prove fruitful.

## Conclusion

Benthic and limnetic stickleback differ in many morphological traits affecting suction feeding, and this functional variation is associated with divergent performance on attached and evasive prey. Ecological divergence in sympatric stickleback involves the evolution of functional divergence via multiple phenotypic traits, and suggests that examining trait utility can provide a fundamental contribution to studies of adaptive radiation.

## Methods

### Collections and photography

We used previously-collected samples from Paxton Lake benthic and limnetic species (Paxton Lake, Texada Island, British Columbia, [17]). A total of 48 fish (benthic n = 23, limnetic n = 25) were used. Fish had been previously fixed in formalin and stored in ethanol; we cleared them in a trypsin solution and stained bones with alizarin red, then placed the specimens into glycerin for measurement [82]. Clearing with trypsin restores a more natural range of motion to the muscles and ligaments than is present in formalin-preserved fish, allowing us to manipulate the head and jaws more effectively.

Photographs of the fish were taken using a Sony DSC-717 5MP camera attached to a dissecting microscope with a Scopetronix microscope adapter. Three photographs were taken of each fish: one of the head in dorsal view, one lateral head shot with the fish’s jaws closed, and one lateral head shot with the jaws fully protruded and head elevated. Each fish’s mouth was opened using a combination of forceps squeezing the fish’s epaxial and hypaxial muscles, which are involved in opening the mouth during a feeding event, and using a small metal rod inserted into the buccal cavity to press dorsally against the ventral surface of the neurocranium, which rotates upward to open the jaws in life. Applying force to the neurocranium rather than to the jaws directly reduces the ability of the investigator to open the jaws farther than they would move in a live specimen. Each photograph also contained a ruler for scale.

### Functional morphology

We used eleven landmarks to measure the morphological components of four functional systems associated with prey capture: the suction index model, the opercular four-bar linkage model, the opening jaw lever system, and jaw protrusion. Ten of the distances between landmarks (hereafter, “component traits”) are then used to calculate 4 key performance traits of the four functional systems using the formulas in [29,32,33,61]. Landmarks were digitized using the MATLAB program DLtdv3 [83], from which linear distances could be calculated between pairs of x and y coordinates. Epaxial width landmarks were measured from the dorsal photographs. Epaxial height, four-bar input link, coupler link, and fixed link landmarks were measured from the closed-mouth lateral photographs. Gape, buccal length, neurocranium outlever, and jaw opening outlever landmarks were measured from the open-mouth lateral photographs. Calipers were used to measure standard length (SL), defined as the distance from the anterior-most point of the closed upper jaw to the posterior-most point of the vertebral column. The distance between the insertion of the interopercular-articular ligament and the point of articulation between the quadrate and articular (landmarks 8 and 9, Table 1), which is used to calculate both opening inlever and the output link of the opercular four-bar, is not in plane in a lateral photograph. We measured this distance by hand in all fish using a dissecting microscope at 50× magnification with an ocular micrometer (r^2^ = ?).

From these measurements we calculated suction index, the displacement advantage (the ratio of output to input displacement) of lower jaw opening, the transmission coefficient of the opercular four-bar for a five-degree input rotation, and jaw protrusion (Figure 2). The transmission coefficient refers to the amount of rotation produced by the output link for a set amount of rotation in the input link [30]. We tested for divergence in these functional traits using Wilcoxon signed-rank tests (Table 1). We also tested for limnetic-benthic differences in the 11 component traits used to calculate the functional traits.

Before analyzing the individual component traits used to derive our functional indices, we corrected each trait for size with a log-log regression on standard length (SL). We chose standard length over other possible size traits (eg. centroid size), because SL is less affected by the functionally important hypertrophied epaxial musculature of benthic stickleback. We used standardized major axis regression in the R package ‘smatr’ to verify there were no statistically significant interactions between species and SL (at alpha = 0.05). In order to size correct our traits, we calculated residuals from a log-log linear regression of each trait on SL and species, then calculated each trait at a common SL of 50 mm. We report the results of tests on these adjusted traits, but tests on the residuals give equivalent results.

### Kinematics

All protocols for animal use and treatment were reviewed and approved by the University of British Columbia Animal Care Committee and were in compliance with the guidelines of the Canadian Council on Animal Care, application number A07-0293. Live fish used in the kinematic analysis came from two experimental ponds at the University of British Columbia. Each pond had been stocked with either wild adult benthic or limnetic fish from Paxton Lake, British Columbia during the previous summer, and fish were allowed to reproduce naturally. Juvenile stickleback were trapped using unbaited minnow traps and transferred to 110 L aquaria. Each fish was then placed singly in a 20×10×9 cm plexiglass container attached to the top edge of the tank. Sex is known to affect stickleback kinematics [84], so we only filmed non-sexually dimorphic juvenile fish. Fish were filmed using a NAC Memrecam ci digital system (Tokyo, Japan) at 500 Hz. We filmed feeding strikes on live cladocerans (*Daphnia magna*), as cladocerans occur in both littoral habitat and open water, benthic and limnetic stickleback both consume cladocerans in the wild, and both species deplete cladoceran populations in mesocosm studies [85]. Prey were introduced to the aquarium singly with a pipette. We filmed until we obtained at least eight full-effort lateral strikes per individual in benthics (n = 5) and limnetics (n = 5). After filming, each fish was euthanized with an overdose of MS-222.

We used a custom modification of the DLTdv3 MATLAB package [83] to digitize and analyze each strike. We tracked ten landmarks on the head and used them to calculate excursion and timing variables for gape, jaw protrusion, cranial rotation, lower jaw rotation, and strike distance as described in Oufiero et al. [86]. Excursion variables record the maximum value of a distance variable, whereas timing variables indicate the time it takes for the fish to reach its maximum for the appropriate excursion variable. We excluded film sequences in which fish exhibited low effort on the strike, defined as a maximum gape less than 75% of the maximum gape recorded for that individual. Once those sequences were excluded, we retained the three sequences for each individual with the fastest time to peak gape, defined as the time in milliseconds between 20% of peak gape and 95% of peak gape. To ensure that sequences filmed from the same individual were not treated as statistically independent, we used linear mixed models to compare kinematics between species. We treated species and SL as fixed effects and each individual fish as a random effect. Including SL as a fixed effect allows us to control for the expected effect of body size on teleost kinematics [87]. We used ‘pvals.fnc’ from the ‘languageR’ R package to perform an MCMC permutation test to estimate p-values and effect size for our fixed effects [88]. 10,000 MCMC samples were generated for each mixed model analysis, and p-values for species were examined for each of the kinematic variables (Table 2).

We calculated the hydrodynamic (suction) force exerted on a simulated attached prey using the Suction Induced Force Field model, SIFF [28]. We parametrized SIFF using our previously-described kinematic data in the same manner as [28], combined with Suction Index measurements. We then used SIFF to calculate the maximum force that would be exerted on a circular 2 mm prey item during each strike, retaining the three highest-force strikes per individual for analysis using the linear mixed-model approach described above.

## Abbreviations

SIFF: Suction induced force field; SL: Standard length.

## Competing interests

The authors declare that they have no competing interests.

## Authors’ contributions

MDM, DS, and PCW designed the study. MDM made all measurements and filmed all fish. MDM wrote the first draft paper, with DS and PCW providing assistance for following drafts. All authors have read and approved the final manuscript.
